# Spike-Timing-Dependent Plasticity With Axonal Delay Tunes Networks of Izhikevich Neurons to the Edge of Synchronization Transition With Scale-Free Avalanches

**DOI:** 10.3389/fnsys.2019.00073

**Published:** 2019-12-04

**Authors:** Mahsa Khoshkhou, Afshin Montakhab

**Affiliations:** Department of Physics, College of Sciences, Shiraz University, Shiraz, Iran

**Keywords:** neuronal network, spike-time dependent plasticity, critical dynamics, time delay, Izhikevich neuron, neuronal avalanches, synchronization, chemical synapses

## Abstract

Critical brain hypothesis has been intensively studied both in experimental and theoretical neuroscience over the past two decades. However, some important questions still remain: (i) What is the critical point the brain operates at? (ii) What is the regulatory mechanism that brings about and maintains such a critical state? (iii) The critical state is characterized by scale-invariant behavior which is seemingly at odds with definitive brain oscillations? In this work we consider a biologically motivated model of Izhikevich neuronal network with chemical synapses interacting via spike-timing-dependent plasticity (STDP) as well as axonal time delay. Under generic and physiologically relevant conditions we show that the system is organized and maintained around a synchronization transition point as opposed to an activity transition point associated with an absorbing state phase transition. However, such a state exhibits experimentally relevant signs of critical dynamics including scale-free avalanches with finite-size scaling as well as critical branching ratios. While the system displays stochastic oscillations with highly correlated fluctuations, it also displays dominant frequency modes seen as sharp peaks in the power spectrum. The role of STDP as well as time delay is crucial in achieving and maintaining such critical dynamics, while the role of inhibition is not as crucial. In this way we provide possible answers to all three questions posed above. We also show that one can achieve supercritical or subcritical dynamics if one changes the average time delay associated with axonal conduction.

## 1. Introduction

Since its inception nearly two decades ago, the critical brain hypothesis has gained a considerable amount of attention in the literature (Legenstein and Maass, 2007; Chialvo, 2010; Plenz, 2014). Although it has encountered some skepticism at times (Beggs and Timme, 2012), it has now grown to a relatively mature field with substantial body of theoretical and experimental evidence to support it (Beggs and Plenz, 2003, 2004; Levina et al., 2007; Plenz and Thiagarajan, 2007; Beggs and Timme, 2012; Friedman et al., 2012; Haimovici et al., 2013; Fontenele et al., 2019). Brain criticality is thought to underlie many of its fundamental properties such as optimal response, learning, information storage, as well as transfer (Kinouchi and Copelli, 2006; Shew et al., 2009; Larremore et al., 2011; Shew and Plenz, 2013; Hesse and Gross, 2014; Gautam et al., 2015; Clawson et al., 2017). The original ideas of brain criticality came out of studies of self-organized criticality, where a threshold dynamics leads to a balance between slow drive and fast dissipation in open nonequilibrium systems and thus observation of critical dynamics (Bak et al., 1987). It is now generally believed that long-term evolution has led to a balance between excitatory as well as inhibitory tendencies which place the brain “on the edge,” i.e., a critical point. However, this does not necessarily answer the problem of stability of the critical state, as some neurophysiological mechanism is needed to maintain the system near the critical point against many possible perturbative effects.

It seems like there are some important theoretical issues which have remained open in regards to brain criticality: (i) What exactly is the phase transition which determines the critical point? Traditionally, this has been assumed to be the absorbing-state phase transition motivated by the studies of self-organized criticality (Montakhab and Carlson, 1998; Vespignani et al., 2000). However, in some recent studies, it has been indicated that the brain is maintained near a synchronization transition (Poil et al., 2012; Gautam et al., 2015; di Santo et al., 2018; Dalla Porta and Copelli, 2019; Fontenele et al., 2019). We note that some authors have also argued for the existence of the extended critical region similar to that of “Griffiths phase” (Munoz et al., 2010; Moretti and Munoz, 2013; Odor et al., 2015; Moosavi et al., 2017). However, such critical regions also typically occur near the absorbing phase transition where the system transitions from an inactive phase to an active phase. (ii) What is the self-organizing mechanism which leads to, and maintains the system in a critical state? As mentioned above the balance between excitatory and inhibitory tendencies are thought to be the long time solution to this question. However, physiological mechanism such as synaptic plasticity are also considered to be important mechanism to maintain the nervous system in a balanced state on shorter time scales. Clearly, extended criticality can also alleviate such a problem to a certain extend as criticality is observed for a range of parameter instead of a particular point. (iii) If the brain is in the critical state with its associated scale-invariant behavior, how could it also display definitive rhythmic behavior via brain oscillations?

Brain plasticity is increasingly being recognized as an important and fundamental property of a healthy nervous system. In particular, spike-timing-dependent-plasticity (STDP) is an important mechanism which can modify synaptic weights on very short time scales. Therefore, it seems reasonable to invoke STDP as a self-organizing mechanism. In a STDP protocol, the strength of a synapse is modified based on the relative spike-timing of its corresponding pre- and post-synaptic neurons, i.e., STDP incorporates the causality of pre- and post-synaptic spikes into the synaptic strength modifications. If the pre-synaptic neuron spikes first and leads to the post-synaptic neuron to spike shortly afterward, then the synapse is potentiated. Reversely, if the pre-synaptic spike follows the post-synaptic spike the synapse will be depressed (Song et al., 2000; Bi and Poo, 2001; Sjostrom and Gerstner, 2010; Markram et al., 2012). The competition between coupling and decoupling forces arising from successive potentiation and depression of synapses tunes the neural network into a balanced dynamical state.

Our work in this paper is motivated by the above considerations. In particular, we propose to study a biologically plausible model of cortical networks, i.e., Izhikevich neurons, along with neurophysiological regulatory mechanism such as STDP with suitable axonal conduction delays in order to answer some of the above posed questions. Interestingly, we find that our regulatory system self-organizes the neuronal network to the “edge of synchronization” in physiologically meaningful parameter regime. We first establish some of the characteristics of such a steady state. More importantly, we look for characteristics of critical dynamics in such a minimally synchronized steady state. Motivated by various experiments, we look for neuronal avalanches, branching ratios, as well as power spectrum of activity time-series. We find that such a system on the edge of synchronization exhibits significant indications of critical dynamics including scale-invariant avalanches with finite-size scaling. Our results provide plausible answers to the above questions in a biologically relevant (microscopic) model of neuronal networks.

In the following section, we describe the model we use for our study. Results of our numerical study is presented in section III, and we close the paper with some concluding remarks in section IV.

## 2. Model and Methods

The studied cortical networks consist of *N* spiking Izhikevich neurons which interact by transition of chemical synaptic currents with axonal conduction delays. The dynamics of each neuron is described by a set of two differential equations (Izhikevich, 2003):

(1) $$\begin{array}{l} \frac{dv_{i}}{dt}=0.04v_{i}^{2}+5v_{i}+140-u_{i}+I_{i}^{DC}+I_{i}^{syn} \end{array}$$

(2) $$\begin{array}{l} \frac{du_{i}}{dt}=a\left(bv_{i}-u_{i}\right) \end{array}$$

with the auxiliary after-spike reset:

(3) $$\begin{array}{l} \text{if }v_{i}\geq 30,\;\text{then }v_{i}\;\to \;c\;\text{and }u_{i}\;\to \;u_{i}+d \end{array}$$

for *i* = 1, 2, …, *N*. Here *v*_*i*_ is the membrane potential and *u*_*i*_ is the membrane recovery variable. When *v*_*i*_ reaches its apex (*v*_*max*_ = 30 mV), voltage and recovery variable are reset according to Equation (4). *a*, *b*, *c*, and *d* are four adjustable parameters in this model. Tuning these parameters, Izhikevich neuron is capable of reproducing different intrinsic firing patterns observed in real excitatory and inhibitory neurons (Izhikevich, 2003). We set these parameters so that excitatory neurons spike regularly and inhibitory neurons produce fast spiking pattern (Izhikevich, 2003, 2006, 2007).

The term $I_{i}^{DC}$ is an external current which determines intrinsic firing rate of uncoupled neurons. Regularly spiking Izhikevich neurons exhibits a Hopf bifurcation at *I*^*DC*^ = 3.78 (Khoshkhou and Montakhab, 2018). We choose values of $I_{i}^{DC}$ randomly from a Poisson distribution with the mean value 10. The term $I_{i}^{syn}$ represents the chemical synaptic current delivered to each post-synaptic neuron *i* (Roth and van Rossum, 2009):

(4) $$\begin{array}{l} I_{i}^{syn}=\frac{V_{0}-v_{i}}{D_{i}}\underset{j}{\sum }g_{ji}\frac{exp\left(-\frac{t-\left(t_{j}+\tau_{ji}\right)}{\tau_{s}}\right)-exp\left(-\frac{t-\left(t_{j}+\tau_{ji}\right)}{\tau_{f}}\right)}{\tau_{s}-\tau_{f}} \end{array}$$

Here *D*_*i*_ is the in-degree of node *i*, *t*_*j*_ is the instance of last spike of pre-synaptic neuron *j*, and τ_*ji*_ is the axonal conduction delay from pre-synaptic neuron *j* to post-synaptic neuron *i*. If axonal delays are not taken into account, then τ_*ji*_ = 0 for all *j* ≠ *i*. Axonal delay values of τ_*ji*_ are chosen randomly from a Poisson distribution with mean value τ = 〈τ_*ji*_〉. τ_*f*_ and τ_*s*_ are the synaptic fast and slow time constants and *V*_0_ is the reversal potential of the synapse. If inhibition is included, then motivated by the properties of cortical networks (DeFelipe, 1993), we set population density of inhibitory neurons to twenty percent, i.e., α = 0.2 while the initial strength of inhibitory synapses are chosen four times the strength of excitatory synapses. Therefore, the excitation-inhibition ratio is balanced. α = 0 indicates that we are only considering a network of excitatory neurons. *g*_*ji*_ is the corresponding element of the adjacency matrix of the network which denotes the strength of synapse from pre-synaptic neuron *j* to post-synaptic neuron *i*. Each type of synapses are initially static and have equal strength. *g*_*ji*_ = *g*_*s*_ if neurons *j* and *i* are connected and the synapse is excitatory, *g*_*ji*_ = 4*g*_*s*_ if neurons *j* and *i* are connected and the synapse is inhibitory, and *g*_*ji*_ = 0 otherwise. When we turn the STDP on, strength of *excitatory* synapses are modified according to a soft-bound STDP rule (Song et al., 2000; Bi and Poo, 2001; Sjostrom and Gerstner, 2010; Markram et al., 2012), while the strength of inhibitory synapses are fixed. If post-synaptic neuron *i* fires a spike at time *t* = *t*_*post*_, then the strength of synapse is modified to *g*_*ji*_ → *g*_*ji*_ + Δ*g*_*ji*_, where:

(5) $$\begin{array}{l} \Delta g_{ji}=\{\begin{array}{cc} A_{+}\left(g_{max}-g_{ji}\right)e^{-\frac{\Delta t-\tau_{ji}}{\tau_{+}}} & \text{if }\Delta t>\tau_{ji}\\ -A_{-}\left(g_{ji}-g_{min}\right)e^{\frac{\Delta t-\tau_{ji}}{\tau_{-}}} & \text{if }\Delta t\leq \tau_{ji} \end{array} \end{array}$$

Here, Δ*t* = *t*_*post*_ − *t*_*pre*_ is the time difference of last post- and pre-synaptic spikes, *A*_+_ and *A*_−_ determine the maximum synaptic potentiation and depression, τ_+_ and τ_−_ determine the temporal extent of the STDP window for potentiation and depression, and *g*_*min*_ and *g*_*max*_ are the lower and upper bounds of synaptic strength. The values of all parameters for Izhikevich neuron, synaptic current and STDP rule are listed in Table 1.

**Table 1:** Values of constant parameters used in this study.

Izhikevich neuron	*a*_*ex*_ = 0.02	*b*_*ex*_ = 0.2	*c*_*ex*_ = −65	*d*_*ex*_ = 8	*a*_*in*_ = 0.1	*b*_*in*_ = 0.2	*c*_*in*_ = −65	*d*_*in*_ = 2
Synaptic current	τ_*f*_ = 0.2	τ_*s*_ = 1.7	*V*_0,*ex*_ = 0	*V*_0,*in*_ = −75				
STDP rule	*A*_+_ = 0.05	*A*_−_ = 0.05	τ_+_ = 20	τ_−_ = 20	*g*_*min*_ = 0	*g*_*max*_ = 0.6		

**Figure 1:**
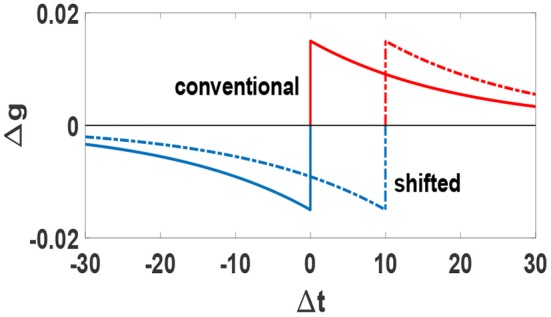
Conventional (solid line) and shifted (dashed line) STDP temporal window function Δ*g* = *f*(Δ*t*). Blue parts denote depression and red parts denote potentiation. Units of Δ*t* is ms.

We consider a temporally shifted STDP window for which the boundary separating potentiation and depression does not occur for simultaneous pre- and post-synaptic spikes, but rather for spikes separated by a small time interval (Babadi and Abbott, 2010). We set the value of this shift equal with the actual axonal delay for each synapse. This rule retrieves the conventional STDP rule when no time-delay is considered, τ_*ji*_ = 0. We have plotted the STDP temporal window function Δ*g* = *f*(Δ*t*) and its shift in Figure 1. This type of time shift introduces a signal transmission time which is more realistic for a causal relation between pre and post synaptic firing. As a result, this temporal shift causes synchronous or nearly-synchronous pre- and post-synaptic spikes to induce long-term depression, which leads to intrinsic stability for the network (Babadi and Abbott, 2010; Asl et al., 2018).

We integrate the dynamical equations using fourth-order Runge-Kutta method with a time step *h* = 0.01 ms and obtain *v*_*i*_(*t*). We typically evolve the entire system for a long time and make sure that the system has reached a stationary state. We then perform our measurements and calculations. We obtain the instants of firings of all neurons and then assign a phase to each neuron between each pairs of successive spikes (Pikovsky et al., 1997):

(6) $$\begin{array}{l} \phi_{i}\left(t\right)=2\pi \frac{t-t_{i}^{m}}{t_{i}^{m+1}-t_{i}^{m}} \end{array}$$

while $t_{i}^{m}$ is the time that neuron *i* emits its *m*^*th*^ spike. We define a time-dependent order parameter:

(7) $$\begin{array}{l} S\left(t\right)=\frac{2}{N\left(N-1\right)}\underset{i\neq j}{\sum }cos^{2}\left(\frac{\phi_{i}\left(t\right)-\phi_{j}\left(t\right)}{2}\right) \end{array}$$

This order parameter measures the collective phase synchronization at time *t*. *S*(*t*) is bounded between 0.5 and 1. If neurons spike out-of-phase, then *S*(*t*) ≃ 0.5, when they spike completely in-phase *S*(*t*) ≃ 1 and for states with partial synchrony 0.5 < *S*(*t*) < 1. The global order parameter S^*^ is the long-time-average of *S*(*t*) at the stationary state after the influence of STDP ($S^{*}={\langle S\left(t\right)\rangle }_{t}$). We note that the intricate details of the model along with the need to obtain long-time dynamics of the system, limit our computational abilities. We have therefore performed simulations for 100 < *N* < 1, 000. We find that our general results and conclusions are independent of the system size used and therefore report most of our results for *N* ≈ 500. In the next section we will present a systematic study of the system above, paying particular attention to the effect of STDP, time delay, and inhibition.

Before we present our results, we note that in our simulations we have also calculated the more common order parameter $R\left(t\right)e^{i\theta }=\frac{1}{N}\underset{j}{\sum }e^{i\phi_{j}\left(t\right)}$ along with the order parameter *S* which we choose to report in this study. Essentially, the same results are obtained for *R* as those obtained for *S*. However, from a statistical point of view *R*(*t*) represents an average of *N* data points while *S*(*t*) represents an average of *N*(*N* − 1)/2 data points which results in better statistics for our limited system sizes, and therefore smoother diagrams given our computational limitations.

## 3. Results

**Figure 2:**
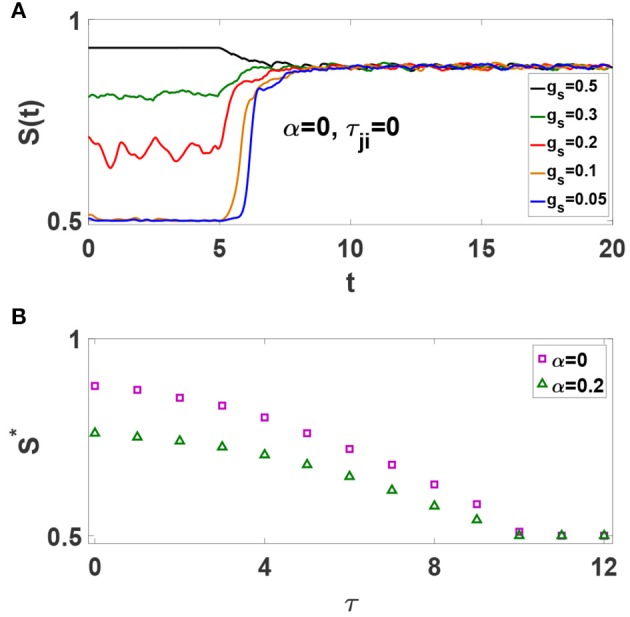
**(A)** Effect of STDP on the time evolution of *S*(*t*) for all-to-all networks of excitatory spiking neurons with different *g*_*s*_. The unit of time axis is in seconds. **(B)** Dependence of S^*^ on τ for α = 0 and α = 0.2.

Spiking Izhikevich neurons with static chemical synapses exhibit a continuous transition to phase synchronization upon increasing synaptic strength, i.e., the amount of global synchrony depends on the average synaptic strength (Khoshkhou and Montakhab, 2018). Now, consider the simple case of an all-to-all network of excitatory neurons without axonal delays. STDP is off initially. *S*(*t*) timeseries for different values of *g*_*s*_ are illustrated in Figure 2A. It is observed that *S*(*t*) depends on *g*_*s*_ as is expected. Next, we turn on the STDP at *t* = 5s. Interestingly, it is seen that *S*(*t*) timeseries evolve to a common state regardless of their initial values. Thus, as STDP modifies the synaptic strengths, neural network organizes into a final state with a specific global phase synchronization S^*^ independent of the initial synaptic strengths. Our investigations reveal that this is a generic condition emerging in neural networks with different underlying structures. We also find that the amount of S^*^ is independent of many parameters including the amplitudes and time extents of STDP rule, and intrinsic firing rate of neurons. However, S^*^ depends drastically on the average value of axonal conduction delays. Figure 2B shows that increasing τ leads to a phase transition from strongly synchronized states with S^*^ ≃ 1 to asynchronous states with S^*^ ≃ 0.5, for neural networks with α = 0 and α = 0.2. Figure 2B also shows that inhibition has a secondary role in the amount of steady state synchronization,S^*^, as compared to axonal delay, τ. Important to our purposes, it shows that for τ = 10 ms the system stands at the boundary of phase synchronization for both α values. Note the importance of time delay as it causes STDP to depress (weaken) the synchronous neurons, thus reducing the amount of S^*^ in the system.

In order to further investigate the properties of Izhikevich neuronal networks, we consider four different networks of *N* = 500: (1) a network of purely excitatory neurons without time-delay (α = 0, τ_*ji*_ = 0), (2) a network of purely excitatory neurons with axonal conduction delays (α = 0, τ = 10 ms), (3) a network of excitatory and inhibitory neurons without time-delay (α = 0.2, τ_*ji*_ = 0), and (4) a network of excitatory and inhibitory neurons with axonal conduction delays (α = 0.2, τ = 10 ms). We have studied networks with different τ values, but we display mostly the results in cases for which all delays are zero (τ_*ji*_ = 0) and S^*^ ≫ 0.5 as well as those with τ = 10 ms for which S^*^ → 0.5^+^. We note that while our results (Figure 2) show that τ = 10 ms is an interesting case of transition point, such an actual value for axonal delay is experimentally meaningful (Swadlow and Waxman, 2012). We turn on STDP at *t* = 5s in a complete network and monitor its influence on different features of each system.

### 3.1. Synchronization and Average Synaptic Weights

**Figure 3:**
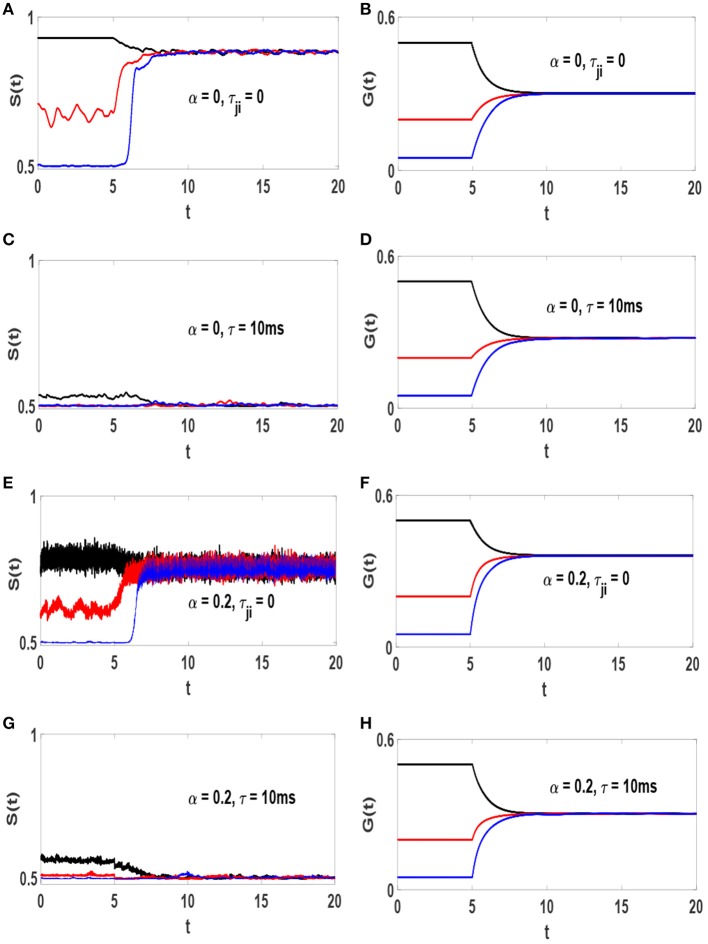
Timeseries of *S*(*t*) and *G*(*t*) and the influence of STDP on them. **(A,B)** α = 0 and τ_*ji*_ = 0, **(C,D)** α = 0 and τ = 10 ms, **(E,F)** α = 0.2 and τ_*j*_*i* = 0, **(G,H)** α = 0.2 and τ = 10 ms. The unit of time axis is in seconds. STDP is turned on at *t* = 5*s*. In each panel different line colors represent different static synaptic strengths, *g*_*s*_ = 0.5 (black), *g*_*s*_ = 0.2 (red) and *g*_*s*_ = 0.05 (blue).

The influence of STDP on the timeseries *S*(*t*) in different conditions is illustrated in the left column of Figure 3. Each panel contains three plots with different values of *g*_*s*_, i.e., the initial synaptic weights. When STDP is off, *S*(*t*) depends on *g*_*s*_. Turning STDP on, each system reaches a final state with a specific amount of synchronization S^*^, regardless of initial level of order (regardless of *g*_*s*_). However, S^*^ depends on τ and α. Systems (1) and (3) reach a strongly synchronized states with S^*^ ≃ 0.88 and S^*^ ≃ 0.75, respectively. Implementation of conduction delays drive the dynamics toward lower levels of order. Systems (2) and (4) with τ = 10 ms lead to states at the edge of transition with S^*^ ≃ 0.509 and S^*^ ≃ 0.503, respectively. The right column of Figure 3 represents the timeseries of the average strength of excitatory synapses, for the corresponding system in the left column represented by, $G\left(t\right)=\frac{1}{N_{L}}\underset{j\neq i}{\sum }g_{ji,ex}\left(t\right)$, where *N*_*L*_ is the number of existing excitatory links. It is observed that at the final states *G*(*t*) ≃ 0.3 for all the systems. It is interesting that the final average value of synaptic weight is independent of the amount of inhibition and and/or axonal delay, as well as initial distribution. However, the main point here is that the amount of synchronization in the system is not solely determined by average synaptic strength but crucially depends on axonal conduction delay, and to a lesser degree on inhibition.

### 3.2. Synaptic Distributions

**Figure 4:**
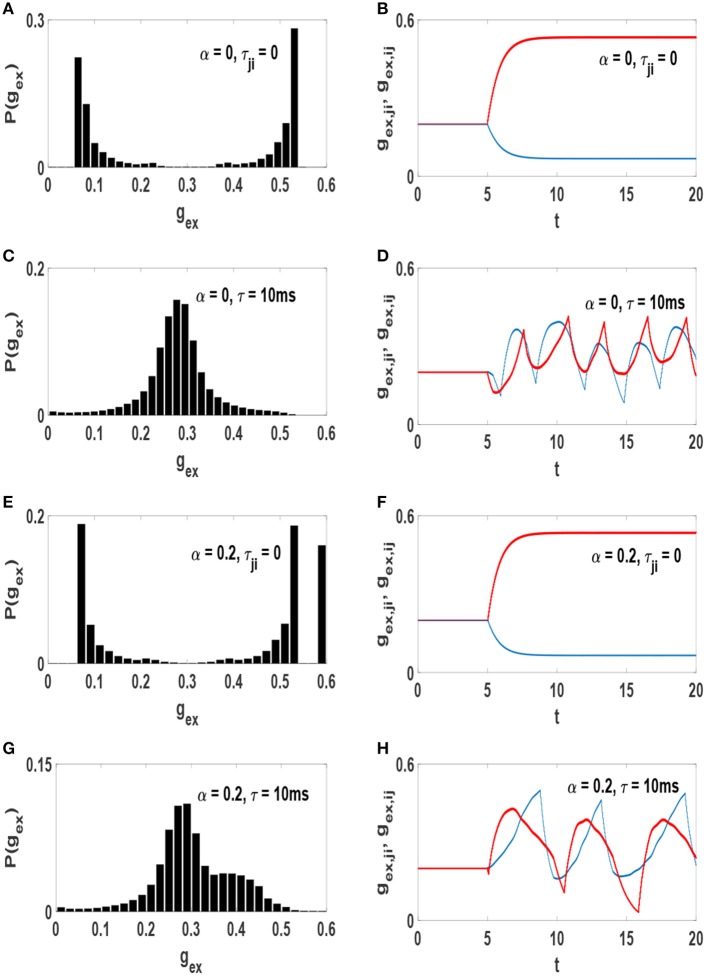
Distribution of the excitatory synaptic strength at the stationary state of the systems after the influence of STDP (left) and time evolution of a pair of reciprocal synapses (right). **(A,B)** α = 0 and τ_*j*_*i* = 0, **(C,D)** α = 0 and τ = 10 ms, **(E,F)** α = 0.2 and τ_*j*_*i* = 0, **(G,H)** α = 0.2 and τ = 10 ms. The unit of time axis is in seconds.

It is somewhat unexpected that similar average synaptic weights would lead to decidedly different synchronization patterns. The answer is in the form of the actual distributions of the weights. In one scenario the average is the most likely value (unimodal distribution) and in the other case is the least likely value (bimodal distribution). The probability distribution function of excitatory synaptic strengths *P*(*g*_*ex*_) (in the steady state) for each system is shown in the left column of Figure 4. Also, the right column of this figure illustrates time evolution of strength of a pair of reciprocal synapses. At the absence of axonal delays, STDP produces a bimodal distribution of synaptic strengths (Figures 4A,E) which is incompatible with the experimentally observed distributions of synaptic strength. However, addition of time-delays to the neural network modifies this condition. Simultaneous presence of STDP and time-delays produce a unimodal distribution of synaptic strengths (Figures 4C,G) resembling those measured in cultured and cortical networks (Turrigiano et al., 1998; Song et al., 2005).

Emergence of these different distributions of synaptic strengths is associated with the amount of phase synchronization in the networks. When neurons interact without time-delay, the final state of the system is strongly synchronized. Therefore, for each pair of symmetric links, STDP depresses the link in one direction and potentiates the link in the opposite direction as in Figures 4B,F). Thus, all symmetric connection would be broken into unidirectional connections after a while in this case. This leads to a bimodal distribution of synaptic strengths whether the network consists of purely excitatory neurons or a mixture of excitatory and inhibitory neurons. With the inclusion of time-delay in the system the level of order declines as it also causes to preserve symmetric connections between each pair of neurons (Asl et al., 2017). Although the strength of synapses fluctuates over time (Figures 4D,H), both links in opposite directions remain active. This leads to a unimodal distribution of synaptic strengths.

### 3.3. Indicators of Criticality

So far we have seen that STDP along with reasonable time delay (and inhibition) will lead the system on the edge of synchronization. However, being on the edge of synchronization could be caused by vastly different spiking patterns (Khoshkhou and Montakhab, 2018). More importantly for the purpose of the present study, we would like to know whether such a state of minimal synchronization has any experimentally relevant indications of criticality. In this subsection we will address such issues.

**Figure 5:**
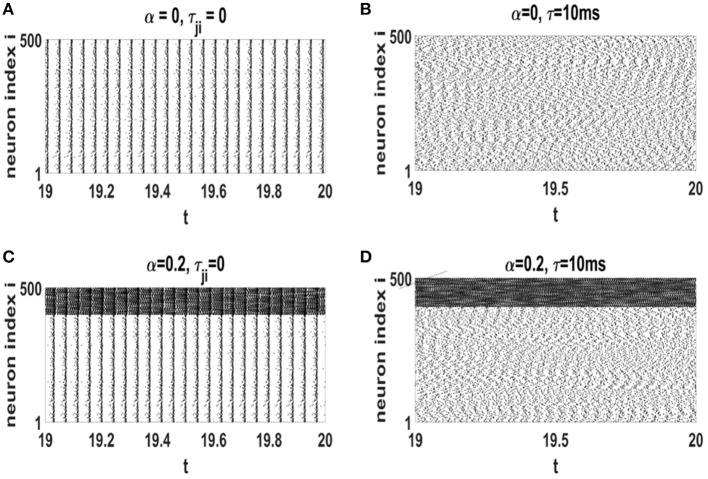
Raster plots of the neural networks with different values of τ and α at the stationary states after influence of STDP. **(A)** α = 0 and τ_*ji*_ = 0, **(B)** α = 0 and τ = 10 ms, **(C)** α = 0.2 and τ_*ji*_ = 0, **(D)** α = 0.2 and τ = 10 ms. The unit of time axis is in seconds.

Raster plots of neural networks with different values of α and τ (in the steady state) are displayed in Figure 5. When time-delay is ignored, neuronal spikes are highly ordered (Figures 5A,C). This is not the real state of a healthy nervous system. However, addition of axonal conduction delay modifies the amount of global order in the networks. Simultaneous effect of STDP and a suitable axonal conduction delays decrease global coherence in neural oscillations (see Figures 5B,D). In Figures 5C,D, inhibitory neurons indexed as 401 − 500, spike with a higher rate as compared to excitatory neurons 1 − 400.

**Figure 6:**
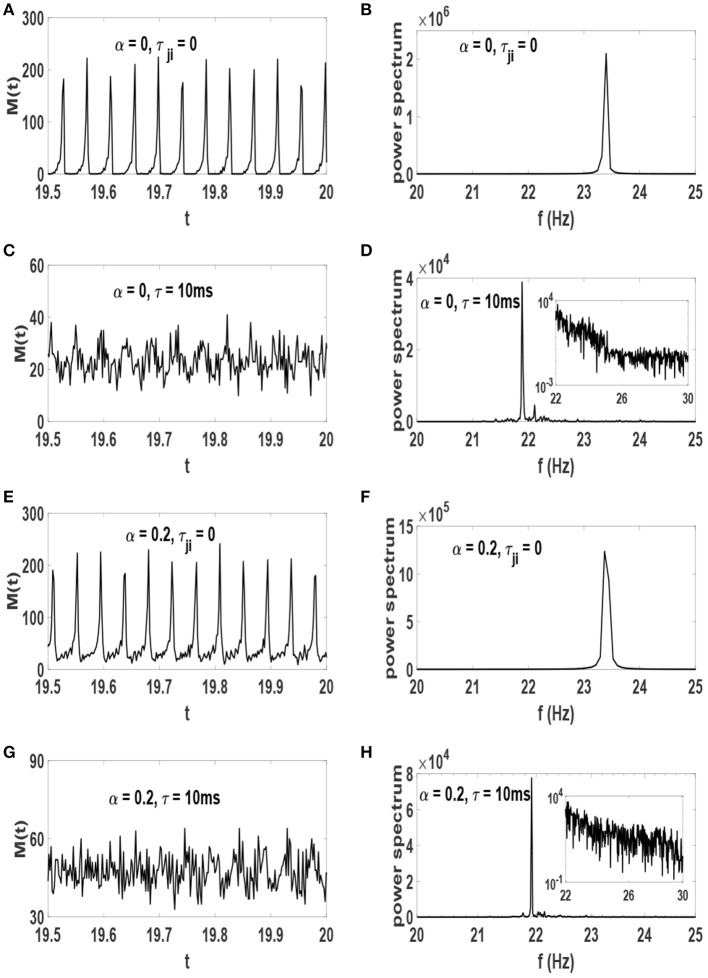
Timeseries of network activity *M*(*t*) in the stationary state after the influence of STDP (left) and their power spectrum (right). **(A,B)** α = 0 and τ_*ji*_ = 0, **(C,D)** α = 0 and τ = 10 ms, **(E,F)** α = 0.2 and τ_*ji*_ = 0, **(G,H)** α = 0.2 and τ = 10 ms. The units of time axis is seconds. The inset of **(D,H)** show the same data on a log-log scale.

The amount of order parameter S^*^ and the raster plots are reasonable evidences showing the system with τ = 10 ms organizes to the edge of synchronization transition point. We now present experimentally relevant results which indicate that such a system is in a critical state. We first consider the network activity timeseries *M*(*t*) which is defined as the number of neuronal spikes at time *t*, as well as its power spectrum. These plots are illustrated in Figure 6. The network activity oscillates regularly in systems without time-delay for which phase synchronization is strong (Figures 6A,E). Therefore, the power spectrum of these systems exhibit a sharp peak at *f* ≃ 23.5 Hz (Figures 6B,F). While neurons are delay-coupled the oscillations of *M*(*t*) are irregular (Figures 6C,G). Despite this deceptive irregularity, the power spectrum exhibits a large peak at frequency *f* ≃ 21.5 Hz (Figures 6D,H) along with a range of other frequencies. This dominant peak reveals that rhythmic oscillations are still robust at these neural networks. The inset of Figures 6D,H show a log-log plot which indicates that the spectrum is fat-tailed in the systems for which τ = 10 ms. Note that the amplitude of oscillations of *M*(*t*) depends on the level of phase synchronization. The stronger the neurons are synchronized, the larger is the amplitude of *M*(*t*) oscillations, i.e., note the scale of the power spectrum on the y-axis.

Scale-invariant statistics of neural avalanches is thought to be the most important indicator of critical brain dynamics. Hence, the network displays spontaneous activity of various sizes *s* and durations *d*, known as neural avalanches, which exhibit scale-free distribution, i.e., $P\left(s\right)\sim s^{-\gamma_{s}}$ and $P\left(d\right)\sim d^{-\gamma_{d}}$ (Beggs and Plenz, 2003). By monitoring the spiking activity of our systems, we can identify outbursts of spikes the number of which is associated with the size, and the lifetime with the duration of avalanches. An avalanche begins when the network activity exceeds a threshold *M*_*th*_ and ends when it turns back below that threshold. Here, we set the threshold to be equal with the mean value of activity in the system. *s* is defined as the total number of spikes during this avalanche, and *d* is the time interval between the onset and offset of the avalanche. Criticality is supposed to be indicated by a power-law behavior and a finite-size cut-off which diverges as system size diverges (*N* → ∞).

**Figure 7:**
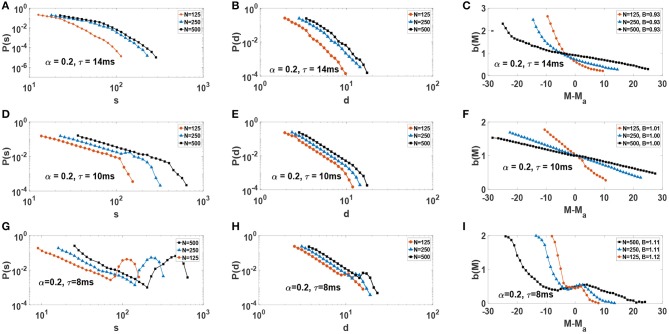
Distribution function of size and duration of avalanches, as well as activity-dependent branching-ratio *b*(*M*) vs. *M* − *M*_*a*_, for various network sizes *N*: **(A–C)** subcritical, **(D–F)** critical, and **(G–I)** supercritical. On the right column, the average branching-ratio *B* for each network with size *N* is reported in the corresponding legend.

We consider neural networks with α = 0.2 and three different τ values, i.e., τ = 14 ms, τ = 10 ms and τ = 8 ms. From the synchronization point of view, Figure 2B, these systems would be subcritical, critical, and supercritical. Each network is also simulated with different network sizes *N*. For any given set of parameters the network is simulated for a considerably long time, producing a large number of avalanches. Probability distribution functions of avalanche sizes and avalanche durations for such networks is illustrated in Figure 7. For neural networks with τ = 14 ms, *P*(*s*) and *P*(*d*) decay with a characteristic scale which is an indicator of subcritical behavior (Figures 7A,B). Note how this scale begins to saturates as system size increases. For networks with τ = 8 ms, *P*(*s*) exhibits a bump for large *s* and *d* which is an evidence of supercritical behavior (Figures 7G,H). Here, large avalanches are more likely to occur than intermediate size avalanches. Interestingly, in networks with τ = 10 ms *P*(*d*) and *P*(*s*) exhibit power-law behavior $P\left(d\right)\sim d^{-\gamma_{d}}$, $P\left(s\right)\sim s^{-\gamma_{s}}$ and a finite-size cut-off which increases relative to the system size (Figures 7D,E).

Another important quantity to characterize critical dynamics is *activity-dependent* branching ratio (Martin et al., 2010). Essentially, this function gives the (relative) expectation value of the timeseries in the next time step for a given amount of activity at the present time step. More precisely, it is defined as, *b*(*M*) = *E*{ξ_*M*_/*M*}. The variable ξ_*M*_ is the value of the next signal given that the present one is equal to *M*, so ξ_*M*_ = {*M*(*t* + *dt*)|*M*(*t*) = *M*} (Martin et al., 2010). Since a critical system is on the edge and is inherently unpredictable, *b*(*M*) ≈ 1, ∀*M*. For a finite system one expects a similar result with the additional consideration that, with increasing system size, the range of activity *M* should increase and that the function should asymptotically approach 1. Therefore, one expects *b*(*M*) < 1 to generally indicate subcritical behavior, while *b*(*M*) > 1 to indicate supercritical behavior. In fact, *b*(*M*) has been used to ascertain criticality in a wide range of systems including sandpile models of SOC or solar flares (Martin et al., 2010) as well as neural networks (Larremore et al., 2014; Moosavi and Montakhab, 2014; Moosavi et al., 2017).

We obtain the activity-dependent branching-ratio *b*(*M*) using timeseries *M*(*t*). The right column of Figure 7 displays *b*(*M*) plots for each one of subcritical, critical and supercritical systems for different system sizes *N* (Figures 7C,F,I). Note that the plots are centered around their respective average activity *M*_*a*_. Only in the critical case (Figure 7F) do we observe *B*(*M*_*a*_) = 1. However, more importantly, we see *b*(*M*) increases its range and decreases its slope (toward zero) with increasing system size, consistent with critical dynamics of the network. In the two other cases, no such behavior is observed. For a more common branching ratio, one calculates the average value of *B*(*M*), i.e., $B=\frac{1}{M_{max}-M_{min}}\int_{M_{min}}^{M_{max}}b\left(M\right)dM$. We find *B* ≃ 1, *B* ≃ 0.93, and *B* ≃ 1.1 again indicating critical, subcritical, and supercritical dynamics accordingly. The average branching ratio, B, is reported on the legend of the corresponding plots in Figure 7 for each system size. However, we emphasize that the behavior of *b*(*M*) is key indicator of criticality in the thermodynamic limit. A close inspection of Figure 7F reveals that the range of activity, *M*, increases with system size, as well as *b*(*M*) becoming increasingly flat. This is a strong indication of *b*(*M*) = 1 for a wide range of possible *M*, for large system sizes.

**Figure 8:**
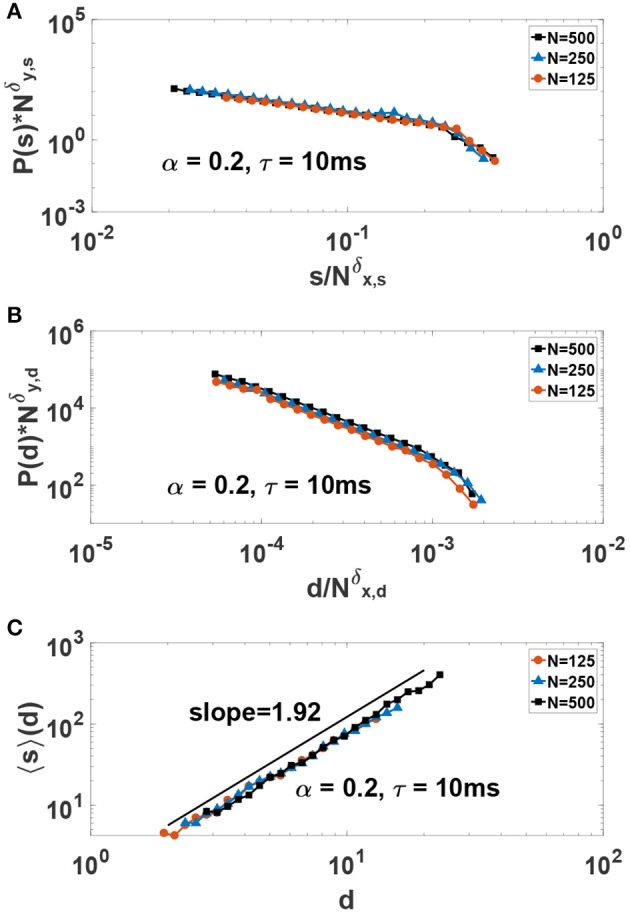
Finite-size-scaling collapse of **(A)** size and **(B)** duration of avalanches in the critical system for δ_*y,s*_ = 1.68, δ_*x,s*_ = 1.16, δ_*y,d*_ = 2.15 and δ_*x,d*_ = 1.14. The critical exponents for avalanche size and duration are γ_*s*_ = δ_*y,s*_/δ_*x,s*_ = 1.45 and γ_*d*_ = δ_*y,d*_/δ_*x,d*_ = 1.88, respectively. **(C)** The average value of avalanches size 〈*s*〉 as a function of avalanches duration *d* in the critical system.

In order to better study the scaling behavior of neuronal avalanches and provide scaling exponents, we use finite-size scaling arguments. In this way, one can scale the y-axis with $N^{\delta_{y}}$ and scale the corresponding x-axis with $N^{\delta_{x}}$ and seek exponents that collapse the data on the same curve. Finding a good collapse is considered a strong indication of true scaling (i.e., in the thermodynamics limit), and the ratio of the exponents give the desired power-law exponent, i.e., γ = δ_*y*_/δ_*x*_. The results for such a study is presented in Figures 8A,B where a good collapse is observed for both size and duration. We obtain the critical exponents γ_*s*_ = 1.45 ± 0.05 and γ_*d*_ = 1.88 ± 0.1. Incidently, these exponents are close to the experimental values obtained in neuronal avalanche studies (Beggs and Plenz, 2003, 2004). Beside standard scaling relations for avalanche size and duration, the theory of critical phenomena predicts another scaling relation between the average size 〈*s*〉 and duration of avalanches, i.e., $\langle s\rangle \left(d\right)\sim d^{\frac{1}{\sigma \nu z}}$, where the critical exponent $\frac{1}{\sigma \nu z}$ is expressed in terms of other critical exponents (Friedman et al., 2012). The key result is that such an exponent must be related to the previous exponents by the relation: Friedman et al. (2012); Dalla Porta and Copelli (2019); Fontenele et al. (2019):

(8) $$\begin{array}{l} \frac{1}{\sigma \nu z}=\frac{\gamma_{d}-1}{\gamma_{s}-1} \end{array}$$

In Figure 8C we show our results for 〈*s*〉(*d*) vs. *d*. We obtain $\frac{1}{\sigma \nu z}=1.92\pm 0.05$. Fulfillment of Equation (8) is considered as another strong evidence of criticality. Here, we obtain $\frac{\gamma_{d}-1}{\gamma_{s}-1}=1.95\pm 0.05$ which is in good agreement with the value $\frac{1}{\sigma \nu z}=1.92\pm \;0.05$.

We note that critical exponents indicating a universality class different from mean-field, i.e., γ_*s*_ = 3/2 and γ_*d*_ = 2, have been reported in recent theoretical and experimental studies of neural avalanches (Scarpetta and de Candia, 2013; Scarpetta et al., 2018; Dalla Porta and Copelli, 2019; Fontenele et al., 2019). The critical exponents we obtained here are reasonably close to the mean-field exponents originally seen in experiments of Beggs and Plenz (Beggs and Plenz, 2003, 2004). One would expect such exponents on general grounds regardless of the dynamics on a small-world network such as ours (i.e., all-to-all network) (Moosavi and Montakhab, 2015). However, we note that the values of our exponents can be effected by various factors including our limited system size *N* = 500. Also thresholding can have an effect on the value of the exponents (Dalla Porta and Copelli, 2019). As indicated above, we have set the threshold to be *M*_*th*_ = 〈*M*(*t*)〉 and we have checked to verify that the exponents do not change significantly within a standard deviation shift of this threshold. However, as in Dalla Porta and Copelli (2019) one can expects the exponents to change considerably if one changes the threshold by a significant amount.

## 4. Concluding Remarks

Recently, the dynamics of adaptive neural networks to a critical state has attracted attention as an interesting mechanism for self-organized criticality (Shew et al., 2015; Virkar et al., 2016; Del Papa et al., 2017; Kossio et al., 2018). In this paper we showed that invoking neurophysiological regulatory mechanisms such as temporally shifted STDP and specific amounts of axonal conduction delays (τ = 10 ms) in a biologically plausible model of cortical networks put the system in a critical state at the neighborhood of synchronization transition point. In this state the system exhibits robust rhythmic behavior along with power-law behavior of various avalanche distribution functions. Furthermore, the behavior of activity-dependent branching-ratio confirms the criticality of system in this state as well. However for smaller or larger values of axonal conduction delays neural networks self-organize into supercritical or subcritical states, respectively. While the state of the network is off-critical, neither the statistics of avalanches nor branching-ratio exhibit the relevant signs of criticality. In this regard, we have taken extra care to establish the existence of true critical behavior in spite of our limited system size.

Coexistence of rhythmic oscillations and scale-invariant avalanches is important for development of cortical layers (Gireesh and Plenz, 2008). Evidence for this coexistence has been found in experimental investigations (Gireesh and Plenz, 2008; Yang et al., 2012). Also in theoretical studies, this phenomenon has been reported to occur as a result of balance between inhibition and excitation (Poil et al., 2012), as well as in a periodically driven SOC model (Moosavi et al., 2018). The neurophysiological mechanisms leading to this intricate dynamics in the cortex is of fundamental importance in neuroscience. Here, we revealed that such intricate dynamics emerges as a result of intrinsic regulatory mechanisms like STDP and axonal conduction delays. More strictly, we obtained self-regulated criticality along with coexistence of rhythmic oscillations and scale invariant activity in a biologically relevant model. However, we note that being on the verge of synchronization transition, our system exhibits certain patterns of regularity in oscillations as exhibited in raster plots in Figure 5D and more precisely shown in the accompanying power-spectrum as in Figure 6H. In fact, real cortical networks display a broad range of frequency such as a heterogenous power-law behavior in the spectrum. Therefore, despite producing relevant oscillations as well as scale-free avalanches, our model does not reproduce a wide range of frequencies in the power spectrum. This can perhaps be alleviated by choosing a more heterogenous range of intrinsic frequencies for the Izhikevich neurons.

We began this paper by posing three open questions regarding the critical brain hypothesis. Our results have provided interesting possible answers to all three questions. (i) The critical point and corresponding phase transition that the brain organizes itself into is not the usual activity and/or absorbing phase transition, but the synchronization phase transition. (ii) The self-organizing mechanism which tunes and maintains the system around such critical point is a standard neurophysiological regulatory mechanism of a temporally shifted STDP. (iii) The existence of individual neuronal oscillations which self-organize to a highly correlated but weakly synchronized collective state is responsible for a dominate oscillatory mode in addition to scale-free fluctuations.

We have studied neural networks with different (small-world) topologies, various initial conditions, as well as various choices of STDP parameters and observed that our results are generally the same upon all such changes. We have also examined that hard-bound STDP leads to similar results, except for the distribution function of synaptic strengths that would be bimodal regardless of all conditions implemented in the neural network. Regarding the importance of inhibition, our results show that inhibition does not significantly alter our conclusions. In fact, we obtain essentially the same critical behavior for the system without inhibition, i.e., α = 0 and τ = 10 ms, as can be expected from similarity of results in various figures with or without inhibition, e.g., Figure 2B.

## Data Availability Statement

All datasets generated for this study are included in the article.

## Author Contributions

AM and MK designed the project, analyzed the results, and wrote the paper. MK carried out the simulations.

### Conflict of Interest

The authors declare that the research was conducted in the absence of any commercial or financial relationships that could be construed as a potential conflict of interest.
